# Distinct roles for REV-ERBα and REV-ERBβ in oxidative capacity and mitochondrial biogenesis in skeletal muscle

**DOI:** 10.1371/journal.pone.0196787

**Published:** 2018-05-03

**Authors:** Ariadna Amador, Sean Campbell, Melissa Kazantzis, Gary Lan, Thomas P. Burris, Laura A. Solt

**Affiliations:** 1 Kellogg School of Science and Technology, The Scripps Research Institute, Jupiter, Florida, United States of America; 2 Department of Immunology and Microbiology, The Scripps Research Institute, Jupiter, Florida, United States of America; 3 Metabolic Core, The Scripps Research Institute, Jupiter, Florida, United States of America; 4 Department of Pharmacology and Physiology, Saint Louis University School of Medicine, St. Louis, Missouri, United States of America; 5 Department of Molecular Medicine, The Scripps Research Institute, Jupiter, Florida, United States of America; Karlsruhe Institute of Technology, GERMANY

## Abstract

The nuclear receptors REV-ERBα and REV-ERBβ have been demonstrated to be core members of the circadian clock and participate in the regulation of a diverse set of metabolic functions. Due to their overlapping tissue expression patterns and gene expression profiles, REV-ERBβ is thought to be redundant to REV-ERBα. Recent work has highlighted REV-ERBα’s role in the regulation of skeletal muscle oxidative capacity and mitochondrial biogenesis. Considering the similarity between the REV-ERBs and the hypothesized overlap in function, we sought to determine whether REV-ERBβ-deficiency presented with a similar skeletal muscle phenotype as REV-ERBα-deficiency. Ectopic overexpression in C2C12 cells demonstrated that REV-ERBβ drives mitochondrial biogenesis and the expression of genes involved in fatty acid oxidation. Intriguingly, knock down of REV-ERBβ in C2C12 cultures also resulted in mitochondrial biogenesis and increased expression of genes involved in fatty acid β-oxidation. To determine whether these effects occurred *in vivo*, we examined REV-ERBβ-deficient mice and observed a similar increase in expression of genes involved in mitochondrial biogenesis and fatty acid β-oxidation. Consistent with these results, REV-ERBβ-deficient mice exhibited an altered metabolic phenotype compared to wild-type littermate controls when measured by indirect calorimetry. This likely compensated for the increased food consumption that occurred, possibly aiding in the maintenance of their weight over time. Since feeding behaviors are a direct circadian output, this study suggests that REV-ERBβ may have more subtle effects on circadian behaviors than originally identified. Furthermore, these data implicate REV-ERBβ in the control of skeletal muscle metabolism and energy expenditure and suggest that development of REV-ERBα versus REV-ERBβ selective ligands may have therapeutic utility in the treatment of metabolic syndrome.

## Introduction

In order to maintain homeostasis and promote survival, the circadian clock coordinates and maintains the 24-hour rhythmicity of various physiological processes, the most evident are the sleep/wake and fasting/feeding cycles[1, 2]. Disruption of the clock has been associated with an increased susceptibility and/or development of sleep and metabolic disorders[1, 2]. Proper circadian function occurs in response to synchronous expression of the molecular machinery of the circadian clock, composed of a central pacemaker in the suprachiasmatic nucleus (SCN) residing in the hypothalamus. While the master clock is entrained by light, almost all cells in the body harbor peripheral clocks that are entrained by both signals from the master clock and environmental cues[3]. This molecular machinery is comprised of core clock proteins that control each other’s expression and function in a transcriptional/translation feedback loop; two transcriptional activators, BMAL1 and CLOCK, heterodimerize and drive the expression of *Cryptochrome* (*Cry1*, *2*) and *Period* (*Per1-3*) [4]. Once Cryptochrome and Period proteins reach a critical threshold, PER:CRY complexes translocate to the nucleus to repress *Bmal1*:*Clock* transactivation[4]. In addition to these core clock components, the nuclear receptors (NR) RORs (α, β, and γ) and the REV-ERBs (α and β) compete to activate and repress, respectively, the transcription of *Bmal1* and *Clock* genes, completing an integral accessory loop which helps maintain circadian rhythmicity[5–7].

The REV-ERBs also participate in the regulation of a diverse set of metabolic processes, thus linking control of our daily rhythms with maintenance of metabolic homeostasis[8]. Both REV-ERBs are ubiquitously expressed with very high expression patterns in the liver, adipose tissue, skeletal muscle, and brain, with both NRs exhibiting circadian patterns of expression[9–11]. The REV-ERBs are unique within the NR superfamily in that they lack the carboxy-terminal tail of the ligand binding domain (LBD called activation function 2 (AF-2, helix 12), which is required for coactivator recognition. As a result, both REV-ERBs are transcriptional repressors, recruiting corepressors such as NCoR in a ligand-dependent fashion. Both REV-ERBs bind to identical DNA response elements (termed RORE/RevREs) either as monomers to an AGGTCA “half site” with a 5’ AT-rich extension, or as homodimers to a DR2 element [direct repeat (DR) AGGTCA sequence with a 2 base pair spacer][12, 13]. The retinoic acid receptor-related orphan receptors [RORs (α, β, and γ)] recognize the same DNA response element and are often coexpressed in the same tissues as the REV-ERBs[14]. Due to the limited availability of genetic models to explore REV-ERBβ’s function, significantly more is known about REV-ERBα’s role in mammalian physiology. As a consequence, REV-ERBβ is considered functionally redundant to REV-ERBα and its role has, by default, been considered almost identical to REV-ERBα’s.

Recent work has demonstrated that REV-ERBα is highly expressed in oxidative skeletal muscle and plays an integral role in mitochondrial biogenesis and oxidative function[15]. Skeletal muscle accounts for ~40% of body mass, more than 85% of total insulin-stimulated glucose uptake, and is one of the most metabolically demanding major mass peripheral tissues[16, 17]. Consequently, skeletal muscle has a significant role in insulin sensitivity and the development of obesity. Due to REV-ERBα’s role in skeletal muscle, REV-ERBα-deficient mice display changes in daily energy expenditure, pre-disposing them to diet induce obesity[18]. Work performed by Ramakrishnan and colleagues also demonstrated that REV-ERBβ was highly expressed in skeletal muscle, regulating genes involved in fatty acid and lipid absorption[17]. Importantly, data presented by Ramakrishnan and colleagues implicated REV-ERBβ in the control of lipid and energy homeostasis in skeletal muscle[17].

Given the overt skeletal muscle phenotype observed in REV-ERBα-deficient mice, coupled with their metabolic abnormalities and the general view held by some in the field that REV-ERBβ is functionally redundant to REV-ERBα, we sought to determine the extent of functional redundancy of REV-ERBβ to REV-ERBα in skeletal muscle and metabolism[19, 20]. Loss of REV-ERBβ resulted in increased mitochondrial biogenesis and genes involved in muscle metabolism both *in vitro* and *in vivo*. Characterization of REV-ERBβ-deficient mice demonstrated a metabolic profile opposite to that of REV-ERBα-deficient animals, including failure to gain weight despite increased food consumption during the day. This increased food consumption correlated with increased utilization of carbohydrates for energy as well as increased energy expenditure during the day. These data indicate that, at least in terms of muscle, REV-ERBβ is not functionally redundant to REV-ERBα, instead playing a distinct role in the regulation of skeletal muscle metabolism. Furthermore, these data suggest development of dissociated REV-ERB modulators (REV-ERBα-specific versus REV-ERBβ-specific) may be beneficial for the treatment of metabolic syndrome.

## Materials and methods

### Animals

The following mouse strains used were either purchased from the Jackson Laboratory and/or were bred at the Scripps Research Institute–Florida (Scripps Florida). Male B6.Cg-*Nr1d1*^*tm1Ven*^/LazJ, stock # 018447 (REV-ERBα^-/-^)[21]; Male and female REV-ERBβ KO mice were generated as previously described[22]. Wild-type (WT) littermates were used as controls for both knock out strains. This study was carried out in accordance with the recommendations in the *Guide for the Care and Use of Laboratory Animals* of the National Institutes of Health. Animal care and experimental protocols used in this study were approved by the Scripps Florida Institutional Animal Care and Use Committee (Assurance number: D16-00726). Mice were housed in groups of 3–5 in 12 h light/12 h dark cycles at 23°C and fed standard chow (Harlan 2920X), unless otherwise stated. Mice had access to chow and water *ad libitum*. Mice were sacrificed by CO_2_ asphyxiation for tissue processing.

### Food intake

Food intake was monitored in normal chow-fed mice using the BioDAQ system (Research Diets) following vendor recommended procedures. The feeding patterns of pre-acclimatized, individually housed mice were continuously recorded for 3 consecutive days. The daily number of bouts, percent of time spent in bouts and total chow consumed were obtained. A bout constitutes a visit or set of visits (no more than 5 seconds apart) to the food hoppers. Each bout has a duration and amount of food obtained during it. Data represent the average day or night intake. Average values are compared by 2-tailed Student’s *t* test to determine significance.

### CLAMS

Mice were individually placed and acclimatized in a Comprehensive Laboratory Monitoring System (CLAMS; Columbus Instruments) at 23°C for 48 h. Afterwards, VO2, VCO2, food intake, and spontaneous locomotor activity were measured for the indicated time periods. Respiratory exchange ratio (RER) and energy expenditure (EE) were calculated using the following equations: RER = VCO2/VO2; EE (kcal/h) = (3.815 + 1.232 X RER) x VO_2_. EE was normalized by lean body mass. Both raw and average values for RER are presented.

### Body composition studies

Total, fat, lean, and fluid mass of mice was measured by Nuclear Magnetic Resonance using the Minispec LF-NMR (Brucker Optics) analyzer.

### Viral production, cell culture, and infection

To generate murine REV-ERBα or REV-ERBβ retroviral vectors, mouse REV-ERB sequences were inserted into the MIGR1 vector (Addgene) using the XhoI site and further screened for orientation. MIGR1 empty vector was used as a control. MIGR1 was a gift from Warren Pear (Addgene, plasmid # 27490)[23]. To knock down REV-ERBβ expression, 3 different shRNAmirs (TransOmic Technologies, Inc.) were inserted, individually into the pLMPd-Ametrine vector[24]. The shRNAmirs used were top-scoring designs from shERWOOD analysis (TransOmic Technologies, Inc.). Each shRNAmir insert was PCR amplified using primers specific for common regions in the flanking miR-30 sequences, which included XhoI and EcoRI sites. pLMPd-Ametrine containing a CD8 shRNA was used as a control.

Plat-E cells (Cell Biolabs, Inc.) were cultured in DMEM containing 10% fetal bovine serum, 2mM L-glutamine, and 1% penicillin/streptomycin at 37°C under standard culture conditions. Plat-E cells were seeded at 350,000 cells per mL in a 10cm dish the day before transfection. 5μg total plasmid DNA was transfected via Fugene6 reagent (Promega) according to manufacturer’s protocol. Viral supernatant was harvested 48 and 72 hours post transfection and stored at -80.

C2C12 myoblasts were obtained from ATCC (Manassas, VA) and grown in Dulbecco modified Eagle’s medium (DMEM; 4.5 g/l D-Glucose; Gibco) supplemented with 10% fetal bovine serum, 2mM L-glutamine, and 1% penicillin (50units/mL)/streptomycin (50ug/mL). Prior to myogenic differentiation, C2C12s were plated at 30,000 cells/mL in 24 well plates and left to adhere overnight. The following day, cells were washed once with PBS and incubated overnight in 1mL total of 50% retrovirus conditioned media and 50% normal culture media containing Polybrene (8ug/mL, Santa Cruz Biotech). 24 hours later, retrovirus was removed and replaced with normal growth media for another 24 hours before myogenic differentiation was induced. One transduction was sufficient to obtain >90% GFP/Ametrine-positive cells. Myogenic differentiation into myotubes was induced after cells reached confluency by adding DMEM supplemented with 2% horse serum (HS), 2mM L-glutamine, and 1% penicillin/streptomycin at 37°C in a humidified incubator under 5% CO_2_ for 6 days. Overexpression and knock down was verified by qPCR analysis.

### MitoTracker and flow cytometry

Retrovirally transduced C2C12 cells were washed with PBS, trypsinized and incubated at 37°C for 20 min with 100nM MitoTracker Red^FM^ dyes (Molecular Probes). MitoTracker Red probe is a red-fluorescent dye that stains mitochondria in living cells and its accumulation is dependent upon membrane potential (Molecular Probes). To measure viability in the samples, a Fixable Viability Dye (Invitrogen/Thermo Fisher) was added to the cultures during the Mitotracker staining step. This dye can be used to stain both live and fixed cells to irreversibly label dead cells prior to analysis. Samples were washed three times in PBS and flow cytometric analysis was performed on a BD LSRII (BD Biosciences) instrument and analyzed using FlowJo software (TreeStar).

### mtDNA quantification

Mitochondrial content was measured using methods previously described[25]. Briefly, genomic DNA was extracted using the Blood & Cell culture DNA Mini Kit (Qiagen) per manufacturer’s instructions from C2C12 samples. DNA was quantified via a standard curve method in order to dilute all samples to 3ng/ul in TE buffer. Once normalized for concentration, samples were subject to RT-qPCR using a cocktail of SYBR green PCR master mix, template DNA, and mtDNA target specific primer pairs, plus H2O. Each sample was run in triplicate and data were averaged. This step was repeated in separate wells using nuclear DNA specific primers and the ratio between mtDNA and nuclear DNA was quantified. Dissociation curves were calculated for all samples to ensure the presence of a single PCR product.

### RNA isolation and quantitative real-time polymerase chain reaction (qRT-PCR)

Muscle was harvested from WT and REV-ERBβ KO mice after a 5 hour fast, at ZT6 (Zeitgeber 6–6 hours into the murine nocturnal period). Total RNA was isolated from tissues by guanidinium thiocyanate/phenol/chloroform extraction[15]. RNA concentrations were adjusted in order to load 500ng of RNA per cDNA reaction. Total RNA was isolated from C2C12 cells using a Quick-RNA MicroPrep kit (Zymo Research) using the manufacturers protocol. RNA concentrations were adjusted in order to load 1μg of RNA per cDNA reaction. cDNA was synthesized using qScript^TM^ cDNA SuperMix synthesis kit (Quanta Biosciences). Quantification of each transcript by quantitative real time-polymerase chain reaction (qRT-PCR) was performed using SYBR Green dye (Quanta Biosciences) to detect dsDNA synthesis, and analyzed using cycling threshold (Ct) values. Relative expression was determined using the ΔΔCT method and normalized to the housekeeping gene 18s. Quantitative RT-PCR was performed with a 7900HT Fast Real Time PCR System (Applied Biosystems) using SYBR Green (Roche) as previously described. Primers were designed using Primer3 (primer3.sourceforge.net). Specificity and validation of the primers were determined using an *In silico* PCR software program (genome.ucsd.edu) and melting curve analysis to eliminate the possibility of primer-dimer artifacts and check reaction specificity. Primer sequences can be found in Table 1.

**Table 1:** List of qRT-PCR primers used in the study.

Gene name	Forward primer (5' - 3')	Reverse primer (5' - 3')
*18s*	GTAACCCGTTGAACCCCATT	CCATCCAATCGGTAGTAGCG
*Acadl*	ATCTTTTCCTCGGAGCATGA	TTTCTCTGCGATGTTGATGC
*Acads*	TGACTTTGCCGAGAAGGAGT	ACTCAGCTCCTCTGGCACAT
*Arntl*	CAGGCTAGCTTGATAGGACAGA	CCAGTGTAGGGGTGACTGTAAAC
*Cd36*	GCGACATGATTAATGGCACA	CCTGCAAATGTCAGAGGAAA
*Clock*	AGAACTTGGCATTGAAGAGTCTC	GTCAGACCCAGAATCTTGGCT
*Cpt1b*	TGTCTACCTCCGAAGCAGGA	GCTGCTTGCACATTTGTGTT
*Cry1*	CACTGGTTCCGAAAGGGACTC	CTGAAGCAAAAATCGCCACCT
*Cry2*	CACTGGTTCCGCAAAGGACTA	CCACGGGTCGAGGATGTAGA
*Fasn*	GCACAGCTCTGCACTGTCTACTAC	ATCCCAGAGGAAGTCAGATGATAG
*Il6*	CATGTTCTCTGGGAAATCGTG	TCCAGTTTGGTAGCATCCATC
*Lpl*	ATCAACTGGATGGAGGAGGAGT	TTCTTATTGGTCAGACTTCCTGCT
*Myh1*	AAGCTTCAAGTTTGGACCCACGGT	TCGGCGTCGGAACTCATGGC
*Myh2*	CGAGGCTGACTCGTCCTGCT	GGGGCAGCCTCCCCGAAAAC
*Myh4*	CCAGGCTGCGGAGGCAATCA	TGCTCGGCCACTCTCCTGCT
*Myog*	CCTTAAAGCAGAGAGCATCC	GGAATTCGAGGCATAATATGA
*Nr1d1*	ACCTTTGAGGTGCTGATGGT	CTCGCTGAAGTCAAACATGG
*mt-Co1*	ACTATACTACTAACAGACCG	GGTTCTTTTTTTCCGGAGTA
*mt-Co2*	AACCATAGGGCACCAATGATAC	GGATGGCATCAGTTTTAAGTCC
*mt-Nd1*	GTTGGTCCATACGGCATTTT	TGGGTGTGGTATTGGTAGGG
*Nr1d2*	TGTGAAAACAGGCAAAACCA	CCCTTACAGCCTTCACAAGC
*Per1*	CGGATTGTCTATATTTCGGAGCA	TGGGCAGTCGAGATGGTGTA
*Per2*	AGCGCAAGGTCCGAGTTAG	GCTGCAACTGGAAATATGAAGC
*Per3*	AACACGAAGACCGAAACAGAAT	CTCGGCTGGGAAATACTTTTTCA
*Ppargc1a*	GGAGCTCCAAGACTCTAGACA	CCAAAGTCTCTCTCAGGTAGC
*Rora*	AACCCGAACCCATATGTGAC	ATGTTCTGGGCAAGGTGTTC
*Scd1*	GGAGACCCCTTAGATCGAGTG	CACTCGAATTACTTCCCACCA
*Srebf1*	AACCTCATCCGCCACCTGCT	CACTGGCACGGGCATCCTTC
*Tpm3*	GTCCCGTTGCCGAGAGATGGA	CAGCACGGGTCTCTGCCTCC
*Tnni1*	GCCTATGCGCACACCTTTG	CGGGTACCATAAGCCCACACT
*Tnni2*	AAATGTTCGAGTCTGAGTCCTAACTG	GCCAAGTACTCCCAGACTGGAT
*Ucp2*	GGCTGGTGGTGGTCGGAGAT	CCGAAGGCAGAAGTGAAGTG
*Ucp3*	ACCTGGACTGCATGGTAAGG	GAGAGCAGGAGGAAGTGTGG

### Statistical analysis

All data are expressed as mean±S.E.M. Statistical analysis was performed using GraphPad Prism6 software. For multiple comparisons, two-way ANOVA was performed. Post-test analysis (ANOVA) was performed correcting for multiple comparisons using the Sidak-Bonferroni method. For all other independent data sets, since the data analyzed were not random, statistical significance was determined using two-tailed unpaired Student’s *t*-tests with no correction for multiple comparisons[26]. ANCOVA analysis was performed using SPSS Statistics (IBM). (N per group per study is indicated in each figure legend). Significance was assessed as follows: *p<0.05, ** *p*<0.01, *** *p*<0.005, **** *p*<0.001.

## Results

### Overexpression of the REV-ERBs drives mitochondrial biogenesis

**Fig 1:**
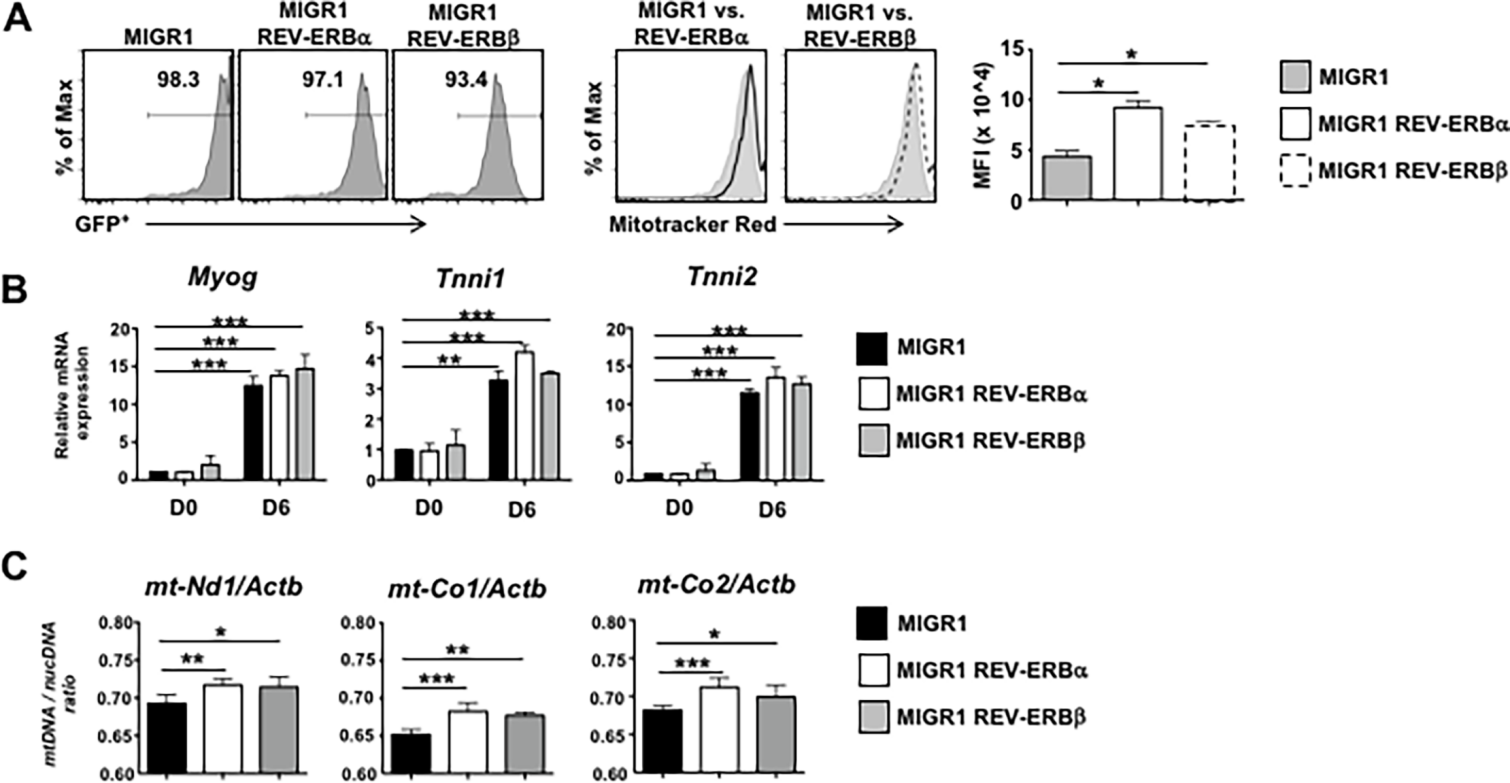
The REV-ERBs modulate mitochondrial content *in vitro*. **(A)** Mitochondrial biogenesis determined by flow cytometry (FACS) using MitoTracker Red staining in empty vector, REV-ERBα, and REV-ERBβ overexpressing C2C12s. FACS panels (left) indicate percent of cells retrovirally transduced. FACS panels (middle) demonstrate overexpression of the REV-ERBs enhances MitoTracker Red staining. Graph (right) depicts median fluorescent intensity (MFI). (n = 3 per condition) **(B)** qRT-PCR analysis of genes demonstrating cells had terminally differentiated. **(C)** Mitochondrial DNA (mtDNA) content demonstrated by the ratio of mtDNA to nuclear DNA (nucDNA) in empty vector, REV-ERBα, and REV-ERBβ overexpressing C2C12s. nucDNA was measured using ß-actin (*Actb*) primers. (n = 3 per condition) Values are mean±s.e.m. Data are representative of 4 independent experiments (Panels A & B) or 2 independent experiments (Panel C) demonstrating similar results. Statistical significance was assessed using Student’s two-tailed *t-*tests. *p<0.05, ** *p*<0.01, ***p<0.001.

We previously demonstrated that REV-ERBα was a critical regulator of muscle mitochondrial biogenesis[15]. To determine the cell autonomous role of REV-ERBβ versus REV-ERBα, we utilized the C2C12 cell line, a well-studied cell culture model commonly used to investigate myogenesis[27–30]. We retrovirally overexpressed REV-ERBα, REV-ERBβ, or empty vector retrovirus in proliferating C2C12 myoblasts two days prior to the induction of differentiation (Fig 1A). MitoTracker Red staining, indicative of functional mitochondria, revealed that overexpression of both REV-ERBs enhanced mitochondrial biogenesis, demonstrated by increased fluorescence in the REV-ERB overexpressing cells relative to empty vector control. Compared to undifferentiated cells (Day 0; D0), the cells had differentiated and acquired a muscle specific phenotype by Day 6 (D6). Very little difference was observed in expression of *Myogenin* (*Myog*), Troponin I slow (*Tnni1*) and Troponin 1 fast (*Tnni2*), at Day 6 (D6) between groups[17] (Fig 1B). No overt viability issues were observed by day 6. (S1A and S1B Fig) Overexpression of both REV-ERBs also resulted in increased mitochondrial content, indicated by the increased expression of mitochondrial NADH dehydrogenase I (*mt-Nd1*), Cytochrome c oxidase I (*mt-Co1*,) and Cytochrome c oxidase II (*mt-Co2*), compared to empty vector control cells (Fig 1C). These data suggest that the REV-ERBs can drive mitochondrial biogenesis *in vitro*.

### Overexpression of the REV-ERBs regulates the molecular clock and mitochondrial metabolism genes

**Fig 2:**
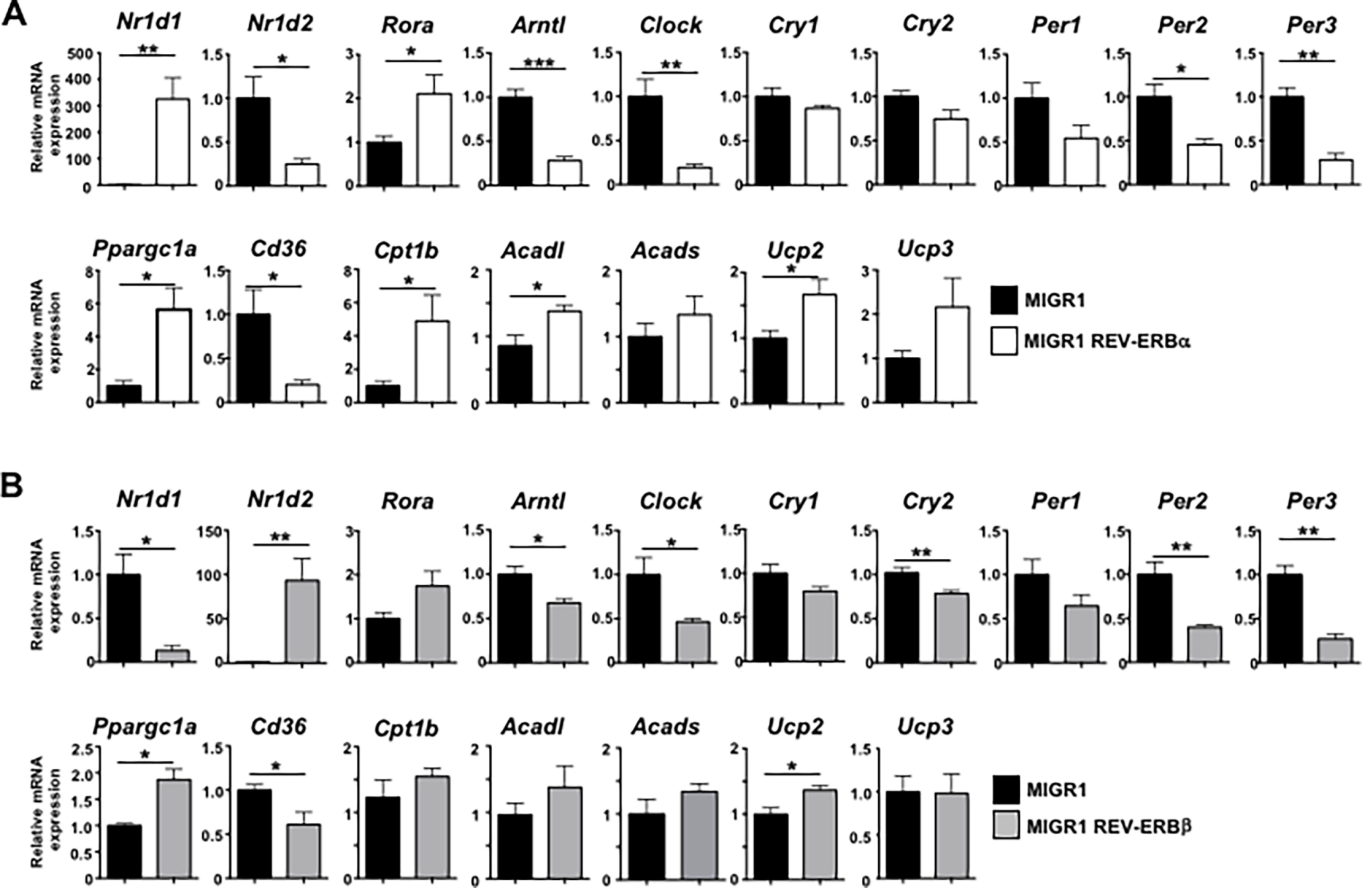
The REV-ERBs modulate circadian and mitochondrial metabolic gene expression *in vitro*. **(A)** qRT-PCR analysis demonstrating that overexpression of REV-ERBα largely represses expression of the molecular clock genes while driving expression of genes involved in mitochondrial metabolism, fatty acid uptake, and fatty acid ß-oxidation. **(B)** qRT-PCR analysis demonstrating that overexpression of REV-ERBβ represses expression of the molecular clock genes while driving expression of genes involved in mitochondrial metabolism, fatty acid uptake, and fatty acid ß-oxidation. *18s* was used as the internal control. Values are mean±s.e.m. Data are representative of 4 independent experiments demonstrating similar results. Statistical significance was assessed using Student’s two-tailed *t-*tests. *p<0.05, ** *p*<0.01.

The REV-ERBs have been demonstrated to be core members of the circadian clock and participate in the regulation of a diverse set of metabolic processes, thus linking control of our daily rhythms with maintenance of metabolic homeostasis[8]. Given the changes in mitochondrial content observed in the REV-ERB overexpressing cells, we next wanted to determine how expression of the clock genes correlated with expression of genes involved in mitochondrial function. Overexpression of REV-ERBα (gene name *Nr1d1*) significantly repressed almost all molecular clock genes, including REV-ERBβ (*Nr1d2*), Bmal1 (*Arntl*), Clock, the Cryptochromes (*Cry1*, *Cry2*), and the Period genes (*Per1*, *Per2*, *Per3*), which is consistent with its role as a transcriptional repressor (Fig 2A). Overexpression of REV-ERBα also repressed expression of *Cd36*, a gene which is involved in lipid uptake[31]. In contrast, RORα and PGC1α (*Ppargc1a*), the master regulator of mitochondrial biogenesis were upregulated with REV-ERBα overexpression. Additionally, genes encoding enzymes of fatty acid β-oxidation, notably carnitine palmitoyltransferase 1b (*Cpt1b*), long chain acyl-CoA dehydrogenase (*Acadl*), short chain acyl-CoA dehydrogenase (*Acads*), *Ucp2*, *and Ucp3*, genes involved in skeletal muscle metabolism, were also upregulated with REV-ERBα overexpression, consistent with previously published work (Fig 2A)[15, 32]. REV-ERBα also repressed expression of *Sterol regulatory element binding-protein 1* (*Srebf1*), *Stearoyl-CoA desaturase-1* (*Scd1*), *Fatty acid synthase (Fasn*), genes involved in lipogenesis, and *Il6*, a pleiotropic muscle myokine that has been shown to be involved in muscle growth, myogenesis, and regulation of energy metabolism (S1C Fig) [33]. Overexpression of REV-ERBβ had similar effects on circadian clock and mitochondrial metabolism genes, with the exception that it potently repressed REV-ERBα expression (Fig 2B). The negative regulation of REV-ERBα on REV-ERBβ expression and vice-versa is consistent their ability to negatively regulate their own expression through conserved ROREs/RevREs in their promoter regions[34, 35]. Interestingly, and in contrast to REV-ERBα overexpression, REV-ERBβ overexpression led to increased expression of *Srebf1* and *Fasn*, but had no effects on expression of *Scd1* or *Il6* (S1C Fig). While it appears that REV-ERBβ was not as effective as REV-ERBα in the overexpression studies, this could be due to the differences in overexpression levels between the two receptors, with REV-ERBα being more highly expressed than REV-ERBβ. Regardless, the increased expression of genes involved in mitochondrial metabolism was consistent with the MitoTracker staining, mitochondrial content, and skeletal muscle metabolism genes. These results demonstrate an inverse relationship between the expression of the core circadian clock genes and the REV-ERBs and indicate that overexpression of REV-ERBα and REV-ERBβ can modulate the expression of genes driving fatty acid β-oxidation and lipid metabolism in C2C12 cells. However, under some circumstances, REV-ERBβ can not completely fulfill REV-ERBα’s role.

### Knock down of REV-ERBβ drives mitochondrial biogenesis

**Fig 3:**
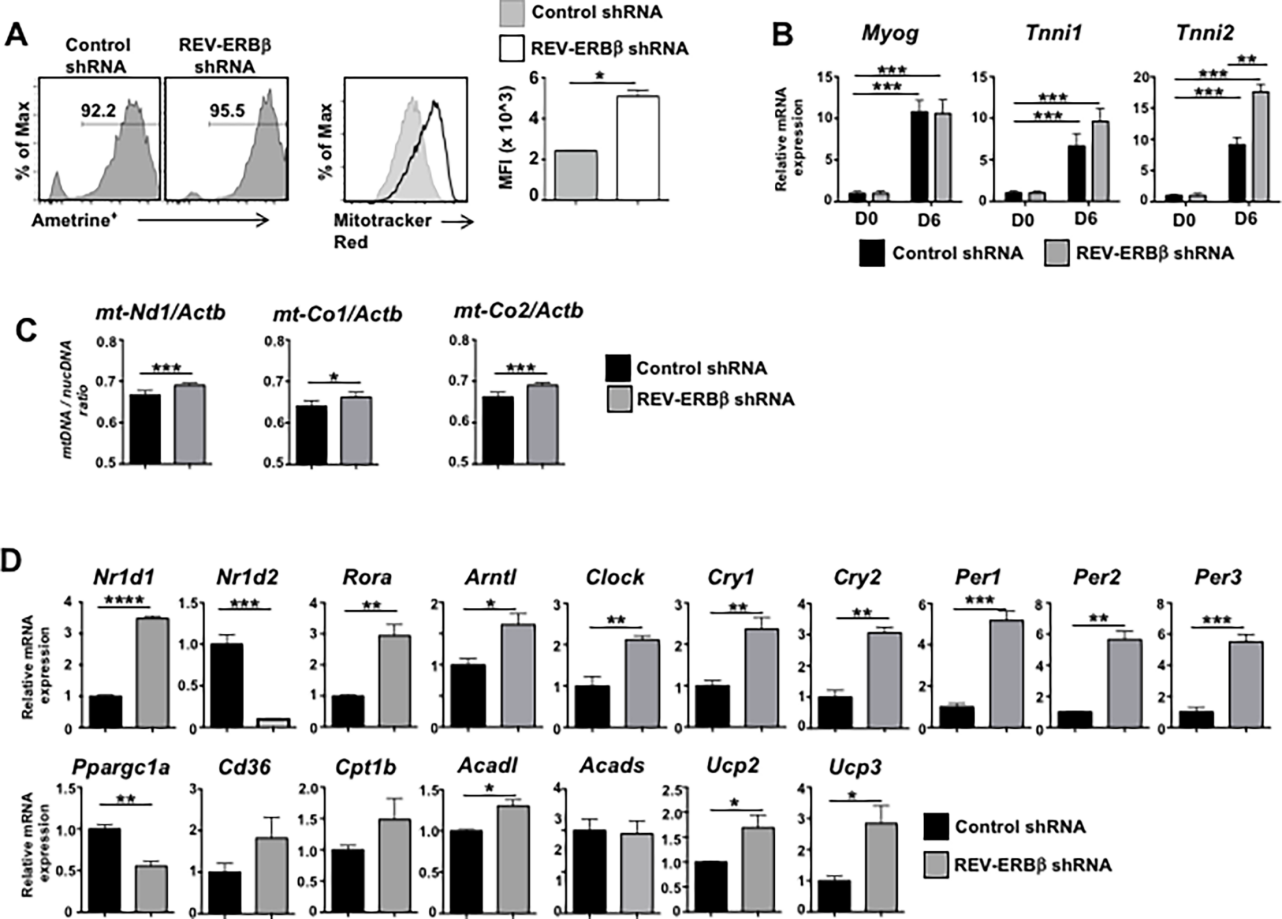
REV-ERBβ knock down drives mitochondrial gene expression *in vitro*. **(A)** Mitochondrial biogenesis determined by flow cytometry (FACS) using MitoTracker Red staining in empty vector and REV-ERBβ shRNA overexpressing C2C12s. FACS panels (left) indicate percent of cells retrovirally transduced. FACS panels (middle) demonstrate knock down of REV-ERBβ enhances MitoTracker Red staining. Graph (right) depicts median fluorescent intensity (MFI). **(B)** qRT-PCR analysis of genes demonstrating cells had terminally differentiated. **(C)** Mitochondrial DNA (mtDNA) content demonstrated by the ratio of mtDNA to nuclear DNA (nucDNA) in CD8 shRNA versus REV-ERBβ shRNA overexpressing C2C12s. nucDNA was measured using ß-actin (*Actb*) primers. **(D)** qRT-PCR analysis demonstrating the knock down of REV-ERBβ and its effects on RORα, REV-ERBα, and genes encoding proteins involved in fatty acid oxidation and cytokine production. *18s* was used as the internal control. (n = 3 per condition). Values are mean±s.e.m. Data representative of 4 independent (Panels A, B, D) or two independent (Panel C) experiments demonstrating similar results. Statistical significance was assessed using Student’s two-tailed *t-*tests. *p<0.05, ** *p*<0.01, *** *p*<0.005, **** *p*<0.001.

Work previously performed assessing REV-ERBβ’s function in C2C12s suggested that it plays a significant role in the regulation of skeletal muscle lipid homeostasis. However, this analysis was performed in C2C12 cells using a dominant negative form of REV-ERBβ that lacked the ligand binding domain[17]. Using this approach, Ramakrishnan and colleagues reported that dominant negative REV-ERBβ largely repressed genes involved in skeletal muscle energy expenditure and lipid catabolism[17]. These results were in line with data demonstrating that loss of REV-ERBα in skeletal muscle led to decreased mitochondrial content and function[15]. Given the overlap in function observed in our overexpression studies, we hypothesized that loss of REV-ERBβ would yield similar results as loss of REV-ERBα. Therefore, to fully assess how loss of REV-ERBβ affects myogenesis, we retrovirally overexpressed a REV-ERBβ shRNA or a CD8 control shRNA in proliferating C2C12 myoblasts 2 days prior to the induction of differentiation (Fig 3A). Strikingly, knock down of REV-ERBβ enhanced mitochondrial biogenesis, indicated by the increased staining with MitoTracker Red. Analysis of *Myog*, *Tnni1*, and *Tnni2* indicated that compared to undifferentiated cells (Day 0, D0), the cells had differentiated and acquired a muscle specific phenotype by Day 6 (D6). No overt viability issues were observed by day 6. (S2A and S2B Fig) Furthermore, loss of REV-ERBβ appeared to significantly increase expression of *Tnni2*. (Fig 3B) Knock down of REV-ERBβ also increased mitochondrial content, indicated by the increased expression of *mt-Nd1*, *mt-Co1*, and *mt-Co2*, compared to CD8 shRNA control cells. Interestingly, while knock down of REV-ERBβ led to increased expression of REV-ERBα and RORα, it also de-repressed all of the core molecular clock genes. Like overexpression of REV-ERBα, overexpression of RORα has also been shown to regulate the expression of genes involved in skeletal muscle metabolism[36]. Knock down of REV-ERBβ also resulted in increased expression of almost all genes analyzed that were involved in lipid and skeletal muscle metabolism, including *Cd36*, *Cpt1b*, *Acadl*, *Ucp2*, *and Ucp3*, genes involved in skeletal muscle, which was consistent with the MitoTracker staining. No change was observed in *Acads*. However, knock down of REV-ERBβ resulted in decreased expression of PGC1α (*Ppargc1a*), *Srebf1*, *Fasn*, and *Il6*, but no change was observed in *Scd1* (Fig 3D and S2C Fig). While these results were largely in contrast to previously published work using a dominant negative REV-ERBβ construct, these results were at least consistent with the notion that in C2C12 cells, REV-ERBβ plays a role in the regulation of muscle metabolism and energy expenditure. Interestingly, these data are in contrast to the overexpression data and show a correlation between the circadian clock and skeletal muscle metabolic gene expression.

### Loss of REV-ERBβ drives skeletal muscle mitochondrial gene expression *in vivo*

**Fig 4:**
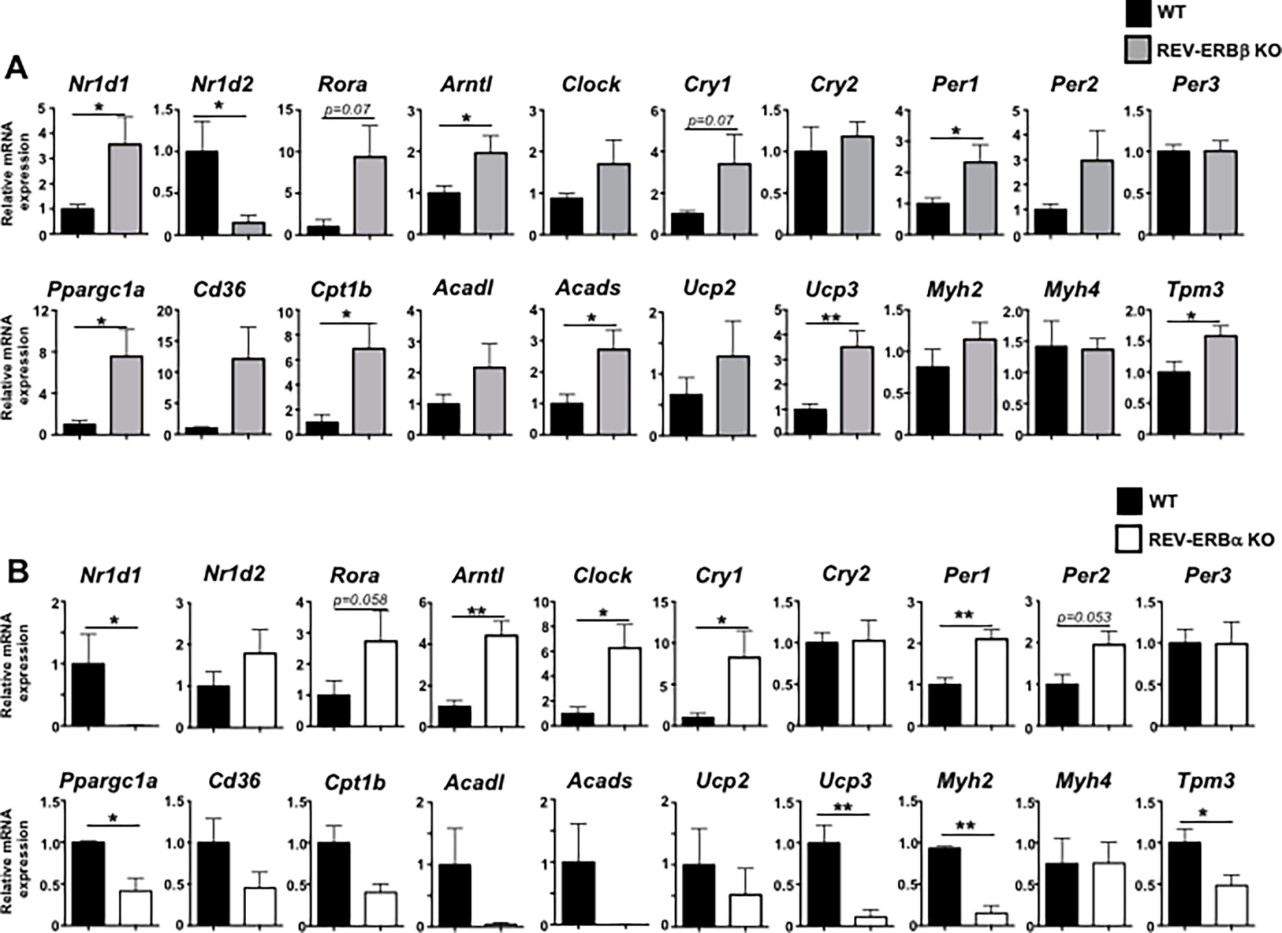
REV-ERBβ-deficient mice show increased muscle expression of mitochondrial and fatty acid oxidation genes compared to REV-ERBα-deficient mice. **(A)** qRT-PCR analysis of muscle (quadriceps) from REV-ERBβ-deficient or wild-type littermate controls. Analysis includes effects on expression on RORα, REV-ERBα, and core molecular clock genes as well as genes encoding proteins involved in fatty acid oxidation, lipid metabolism, and effects on expression on muscle fiber type (*Tpm3*, *Myh2*, *Myh4*). (n = 7 mice per group) Data representative of 3 independent experiments demonstrating similar results. **(B)** qRT-PCR analysis of muscle (quadriceps) from REV-ERBα-deficient or wild-type littermate controls. Analysis includes effects on expression on RORα, REV-ERBα, and core molecular clock genes as well as on expression of genes encoding proteins involved in fatty acid oxidation, lipid metabolism, and expression on muscle fiber type (*Tpm3*, *Myh2*, *Myh4*). (n = 5 mice per group) Data representative of 2 independent experiments demonstrating similar results. Values are mean±s.e.m. Statistical significance was assessed using Student’s two-tailed *t-*tests. *p<0.05, ** *p*<0.01.

Given the surprising results we observed in C2C12 cells, we next wanted to determine whether loss of REV-ERBβ affected skeletal muscle gene expression *in vivo* in a similar manner. Using REV-ERBβ-deficient mice[22], we isolated (whole quadriceps) skeletal muscle from wild-type (WT) and REV-ERBβ knock out (KO) mice at Zeitgeber 6 (ZT6), 6 hours post lights-on and the time point in which peak REV-ERB mRNA expression occurs[37]. qRT-PCR analysis indicated that, similar to the C2C12 data, loss of REV-ERBβ de-repressed most of the core clock genes, including REV-ERBα, RORα, Bmal1, and Clock. We observed no effects on *Cry2* or *Per3* (Fig 4A). Similar results were observed in REV-ERBα KO muscle. (Fig 4B) Loss of REV-ERBβ also led to increased expression of genes involved in lipid and skeletal muscle metabolism (*Cd36*, *Cpt1b*, *Acadl*, *Acads*) and energy expenditure (*Ucp2*, *Ucp3*) (Fig 4A). However, unlike the C2C12 cells, REV-ERBβ KO mice also had increased expression of PGC1α (*Ppargc1a*). Similar to the C2C12s, REV-ERBβ KO mice also exhibited decreased expression of *Srebf1*, *Fasn*, and *Il6*, with little effect observed on *Scd1*. (S3A Fig) Moreover, loss of REV-ERBβ appeared to shift the muscle towards a more oxidative phenotype as we observed increased expression of tropomyosin 3 (*Tpm3*), a marker of oxidative type 1 fiber and myosin heavy chain IIa (*Myh2*), a myosin heavy chain gene more associated with oxidative fibers than others. The expression of *Myh4*, a marker of type 2b glycolytic fibers, was unaltered (Fig 4A). The shift towards a more oxidative phenotype in REV-ERBβ KO mice was in stark contrast to what was observed in REV-ERBα KO mice, which was largely a shift towards a less oxidative phenotype demonstrated by decreased expression of PGC1α, *Cpt1b*, *Acadl*, *Acads*, *Ucp2*, *Ucp3*, *Myh2*, and *Tpm3* and is consistent with previously published results[15]. (Fig 4B) However, not all changes in gene expression between REV-ERBβ KO and REV-ERBα KO mice were conflicting. REV-ERBα KO mice also demonstrated decreased expression of the lipogenic genes *Srebf1* and *Scd1* in muscle while loss of REV-ERBα de-repressed *Fasn* and *Il6* (S3B Fig) However, unlike the muscle tissue, gene expression analysis of livers from REV-ERBβ KO and REV-ERBα KO mice demonstrated similar trends in gene expression (*G6Pase*, *Cd36*, *Clock*) or no effects (*Pepck*) on gene expression *in vivo* (S3C and S3D Fig). These data are consistent with previously published work indicating that REV-ERBα and REV-ERBβ have largely overlapping roles in liver metabolism, with REV-ERBα acting as the dominant factor[19, 20]. These data suggest that in liver, REV-ERBβ appears to have overlapping functions to REV-ERBα. However, in skeletal muscle, the effects mediated by the loss of REV-ERBβ are in contrast to those of REV-ERBα.

### REV-ERBβ-deficient mice exhibit an altered circadian metabolism and feeding schedule

**Fig 5:**
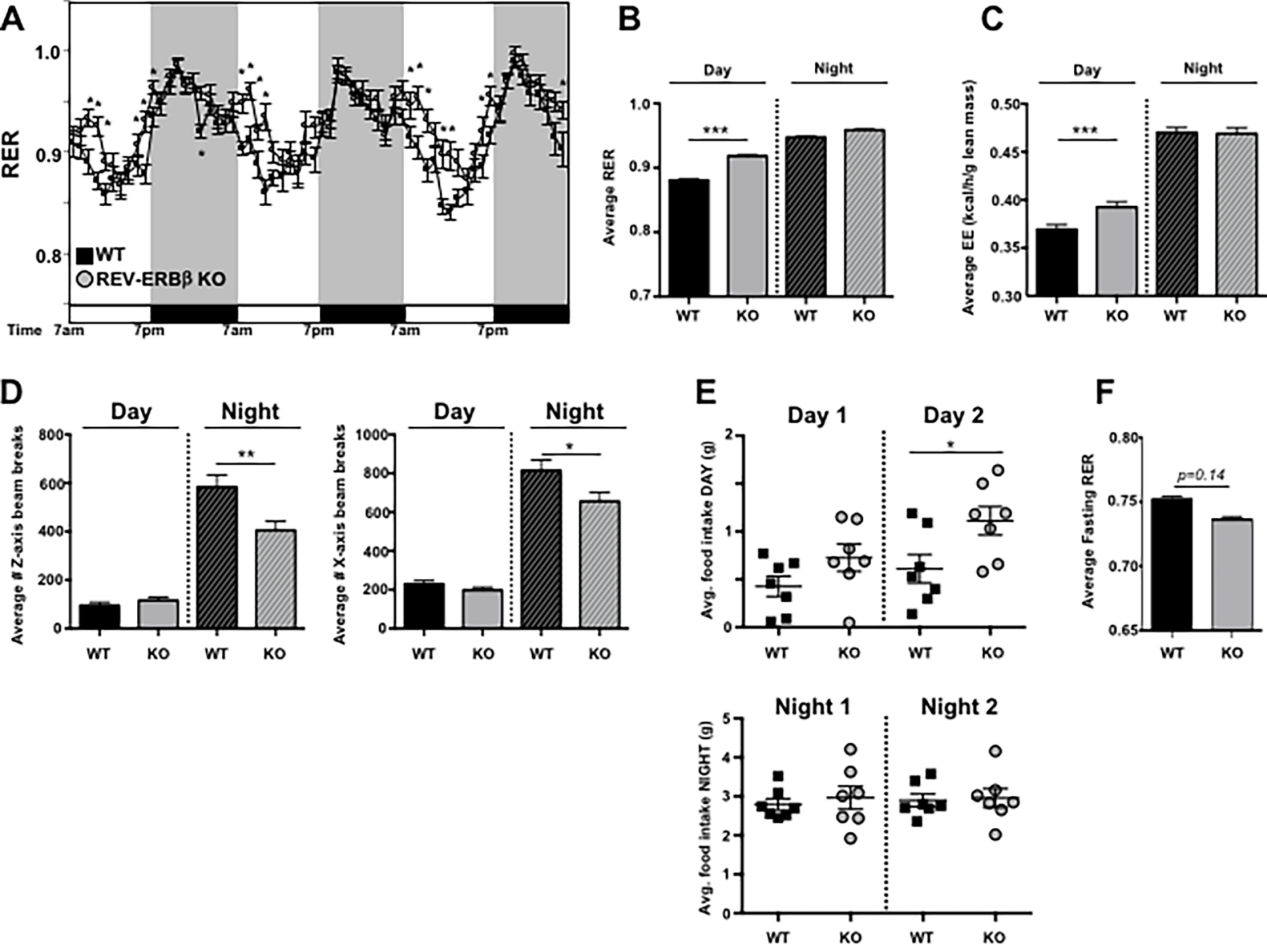
REV-ERBβ-deficient mice exhibit an altered metabolic phenotype and feeding schedule. **(A)** Real-time respiratory exchange ratio (RER) monitoring of pre-acclimated male WT (black square) versus REV-ERBβ KO mice (gray circles) using indirect calorimetry. Mice were fed normal chow over a 3 day period. (F(380,4568) = 5.475, p<0.0001). **(B)** Average RER value, **(C)** energy expenditure, and **(D)** infrared activity beam break counts, indicating movement, for day versus night of the mice in panel A. (n = 7 per group). **(E)** Food intake measurements of male WT (black square) versus male REV-ERBβ KO mice (gray circles) fed normal chow over a 2 day period. **(F)** Average fasting RER value taken from a 6 hour period during the fast. (n = 7 per group) Data are representative of 2 independent experiments demonstrating similar results. Experiments using female mice demonstrated similar results (data not shown). Values are mean±s.e.m. Average RER was obtained using ANCOVA (analysis of covariance)[38] with ambulatory activity used as a covariate. Statistical significance was assessed using two-way ANOVA or Student’s two-tailed *t-*tests. * p<0.05, ** *p*<0.01, *** *p*<0.005.

Due to the increased expression of genes involved in mitochondrial biogenesis in the REV-ERBβ KO mice compared to WT controls, we wanted to determine whether this translated to effects on metabolism and any other circadian driven behavior. Real-time oxygen consumption (VO_2_), carbon dioxide production (VCO_2_), ambulatory activity and food consumption were monitored in twelve week-old male mice fed a normal chow diet using the CLAMS system. Energy expenditure (EE) and RER (Respiratory exchange ratio) were calculated based on the gas exchange as described in the Methods section (Fig 5A–5C). Body composition was obtained just prior to the CLAMS analysis, with no differences observed in lean, fat, and fluid masses or total body weight between groups (S4A Fig). The RER is a surrogate indicator of the caloric source being utilized for energy production by an animal. It varies between 0.7, where lipids are the predominant fuel source, to 1.0, where carbohydrates are the main contributors. The REV-ERBβ KO mice showed a delay in the typical circadian drop in RER that occurs from the dark to light phase transition when mice shift from high activity and feeding to resting and sleep (Fig 5A and 5B). This prolonged high RER cycle indicates an increased relative contribution of carbohydrates as an energy source in the KO animals. This could be due to their increased chow consumption, observed during the light phase while in the CLAMS (S4B Fig). This light-phase specific increase in eating was further confirmed in REV-ERBβ KO mice tested in the BioDAQ system, which uses highly sensitive feeding monitoring hoppers adapted to regular housing cages (Fig 5E, S4D Fig). Additionally, an increase in energy expenditure was detected in association with the observed increased daytime chow consumption in the REV-ERBβ KO mice (Fig 5C). Nighttime energy expenditure in REV-ERBβ KO mice was comparable to WT mice, despite the KO animals being less active during this time (Fig 5D). Whether the increase in energy expenditure in the KO mice during the daytime can be attributed to elevated feeding and digestive processes *per se* remains to be tested. Ambulatory activity during the light-phase was similar between the groups and thus, cannot explain that. However, upon fasting, the KO’s demonstrated decreased RER relative to WT controls, which may be a result from a faster transition to fat burning (Fig 5F). Regardless, the increased caloric output can, at least in part, account for the absence of weight gain by the KO’s in spite of their higher caloric intake (S4E Fig). These data indicate that REV-ERBβ regulates circadian behaviors and metabolism *in vivo*.

## Discussion

REV-ERBα has recently been identified as a key regulator of skeletal muscle mitochondrial function[15]. Due to the overlapping gene expression profiles and similarity in DNA binding domains, REV-ERBβ is thought to be redundant to REV-ERBα. Our studies were aimed at gaining a better understanding of REV-ERBβ’s function in skeletal muscle and overall metabolism. In this report, we utilized the C2C12 *in vitro* cell culture model and REV-ERBβ-deficient mice to investigate the role of this NR in comparison to REV-ERBα’s function in skeletal muscle *in vitro* and *in vivo*. Here we report that overexpression and knock down of REV-ERBβ drives mitochondrial biogenesis in C2C12 cells, exhibited by increased MitoTracker Red staining and increased expression of genes involved in skeletal muscle energy metabolism. We also showed that skeletal muscle from REV-ERBβ KO mice exhibited a similar gene expression profile as the C2C12 cells in which REV-ERBβ was knocked down, which was in contrast to skeletal muscle gene expression in REV-ERBα KO mice. Furthermore, we demonstrated that REV-ERBβ KO mice present with an altered metabolic phenotype and feeding behavior schedule, with KO mice eating more and utilizing more carbohydrates as fuel during their nocturnal/sleep period. In terms of muscle and energy expenditure, our data suggest that REV-ERBβ may not be as functionally redundant to REV-ERBα as originally hypothesized.

Our initial hypothesis was based on the view, held by some in the field, that REV-ERBα and REV-ERBβ are functionally redundant, with REV-ERBα acting as the dominant factor[20]. Overexpression studies are excellent tools to determine whether a protein, in this case REV-ERBβ, is able to perform certain cellular functions. Our initial studies in C2C12 cells supported this hypothesis as overexpression of each REV-ERB resulted in relatively similar functional outputs, largely increased mitochondrial biogenesis and expression of genes involved in skeletal muscle metabolism. However, the shRNA knock down of REV-ERBβ drove further inquiry into REV-ERBβ’s function in skeletal muscle. The increased MitoTracker Red staining and the changes in gene expression observed surprised us, particularly since evaluation of C2C12 cells using a dominant negative REV-ERBβ yielded opposite results to ours[17]. However, this construct still possessed REV-ERBβ’s N-terminal region, which has been shown to interact with the transcriptional co-factor Tip60 in order to regulate gene transcription in a ligand-independent manner[39, 40]. Thus, REV-ERBβ was not completely eliminated and could have recruited co-factors to modify gene transcription in a ligand-independent manner. Alternatively, since RORα and REV-ERBα bind the same DNA consensus sequence as REV-ERBβ, the overexpression of the dominant negative REV-ERBβ could have blocked the ability of RORα or REV-ERBα to bind to the RORE and again, affect transcription. However, use of REV-ERBβ shRNA bypassed these issues and may account for the differences.

Overexpression of REV-ERBα has been demonstrated to drive mitochondrial biogenesis whereas loss of REV-ERBα has the opposite effect[15]. However, the effects rendered by overexpression and loss of REV-ERBβ were not opposite to each other. It is possible that the increased mitochondrial biogenesis in the absence of REV-ERBβ is a consequence of increased REV-ERBα or increased RORα. Indeed, we consistently observed increased expression of both NRs in the absence of REV-ERBβ. Like overexpression of REV-ERBα, overexpression of RORα has also been shown to regulate the transcription of genes involved in lipid metabolism and energy metabolism in skeletal muscle[36]. REV-ERBα, RORα, and REV-ERBβ have been demonstrated to bind to conserved ROREs/RevREs in REV-ERBα’s promoter region[34, 35], with RORα driving gene expression whereas the REV-ERBs repress REV-ERBα gene expression. This autoregulation is likely the reason for the dramatic downregulation of gene expression observed on REV-ERBβ when REV-ERBα was overexpressed and vice versa. It is possible that knock down or loss of REV-ERBβ relieved repression on REV-ERBα, hence the increased expression *in vitro* and *in vivo*. Another possibility is that the overexpression of RORα, which can also bind ROREs, drove gene expression and thus, increased mitochondrial biogenesis. Alternatively, loss of REV-ERBβ could have relieved repression on *Bmal1*, which is known to activate RORα and REV-ERBα transcription through binding to their respective E-box regions[41]. Increased *Bmal1* may account for the increased expression of REV-ERBα and RORα observed both *in vitro* and *in vivo*. Future studies need to be performed to determine the mechanism and which NRs may be driving the increased mitochondrial function in both REV-ERBβ-deficient C2C12 cells and in skeletal muscle *in vivo*.

The circadian clock influences a broad range of physiological processes, including fasting/feeding cycles[2]. Initial analysis of REV-ERBα KO versus REV-ERBβ KO mice indicated that while loss of REV-ERBα affected circadian output, loss of REV-ERBβ had minimal effect[19]. However, our data indicate that REV-ERBβ does affect circadian gene expression in a manner similar to REV-ERBα. Overexpression and knock-out of REV-ERBβ generated a similar gene expression profile as REV-ERBα overexpression and knock-out. However, our data indicate that at least *in vitro*, the circadian clock is not as intimately linked to skeletal muscle metabolism as initially thought. While overexpression and knock down had different effects on expression of core clock components, the skeletal muscle metabolic genes were consistently upregulated and appeared to depend more on the expression of RORα and the REV-ERBs. While this could also be said for the *in vivo* data, we do know that what occurs *in vivo* is significantly more complex and REV-ERBα KO mice present with altered circadian rhythms, which could affect the muscle phenotype[7, 15]. Our data also indicate that REV-ERBβ affects circadian outputs, indicated by increased food consumption during the day. This increased food intake could account for the shift and increased RER and EE observed during the day when measured by indirect calorimetry. However, research has revealed that disordered eating, or eating when we are supposed to be sleeping, contributes to weight gain, metabolic syndrome, and obesity[42, 43]. Despite this, REV-ERBβ KO mice do not gain weight over time, instead maintaining similar weight profiles as WT littermate controls. It is possible that the metabolic demands of the skeletal muscle may compensate for the increased food intake. This may also explain the decreased activity observed in the REV-ERBβ KO mice during their “active” periods. That is, moving less compensated for the increased metabolism with no change in food intake. Alternatively, the food intake during the day may have disrupted their sleep schedule and the decreased movement may be a function of sleep compensation. Clearly, further in-depth analysis of these behaviors is needed to better understand the phenotype observed.

## Conclusions

REV-ERBβ plays a role in skeletal muscle mitochondrial biogenesis both *in vitro* and *in vivo*. This effect on mitochondrial gene expression in REV-ERBβ KO mice differs from that of REV-ERBα KO mice as each receptor appears to have effects opposite to each other. Furthermore, these differences translate to profound differences on metabolism *in vivo*. Our data suggest that REV-ERBβ may not be as functionally redundant to REV-ERBα as originally hypothesized. Thus, development of dissociated REV-ERB modulators (REV-ERBα-specific versus REV-ERBβ-specific) may be beneficial for the treatment of metabolic syndrome.

## Supporting information

S1 Fig Overexpression of the REV-ERBs does not affect C2C12 viability but exerts differential effects on genes involved in lipid metabolism. **(A)** Flow cytometry (FACS) after 6 days in culture using a viability dye. FACS panels (top) are representative plots of the overall health of the C2C12 cultures on Day 6. Graph (bottom) represents the average viability across all cultures per condition (n = 3). **(B)** Representative images of the C2C12 cultures at 10x and 20x magnification on Day 6 demonstrating the differentiation of the cells and no overt cell death. **(C)** qRT-PCR analysis of genes involved in lipid metabolism and inflammation, including *Srebf1*, *Scd1*, *Fasn*, *and Il6* in C2C12 cells overexpressing REV-ERBa (top) or REV-ERBß (bottom) relative to empty vector control. Data are representative of 3 separate experiments demonstrating similar results. Statistical significance was assessed using Student’s two-tailed *t*-tests. *p<0.*05*.
(TIFF)

S2 Fig Knock-down of REV-ERBβ does not affect C2C12 viability but inhibits expression of genes involved in lipid metabolism. **(A)** Cell viability determined by flow cytometry (FACS) after 6 days in culture using a viability dye. FACS panels (top) are representative plots of the overall health of the C2C12 cultures on Day 6. Graph (bottom) represents the average viability across all cultures per condition (n = 3). **(B)** Representative images of the C2C12 cultures at 10x and 20x magnification on Day 6 demonstrating the differentiation of the cells and no overt cell death. **(C)** qRT-PCR analysis of genes involved in lipid metabolism and inflammation, including *Srebf1*, *Scd1*, *Fasn*, *and Il6* in C2C12 cells in which REV-ERBβ was knocked down relative to CD8 control. Data are representative of 3 separate experiments demonstrating similar results. Statistical significance was assessed using Student’s two-tailed *t*-tests. *p<0.*05*.
(TIFF)

S3 Fig REV-ERBβ-deficient mice show differential expression of lipid metabolism genes in muscle compared to REV-ERBα-deficient mice, but not liver. **(A)** qRT-PCR analysis of muscle (quadriceps) from REV-ERBβ-deficient or wild-type littermate controls. Analysis includes effects on expression of genes involved in lipid metabolism and inflammation, including *Srebf1*, *Scd1*, *Fasn*, *and Il6*. **(B)** qRT-PCR analysis of muscle (quadriceps) from REV-ERBα-deficient or wild-type littermate controls. Analysis includes effects on expression of genes involved in lipid metabolism, including *Srebf1*, *Scd1*, *Fasn*, *and Il6*. **(C)** qRT-PCR analysis of genes involved in glucose metabolism (*G6pase*, *Pepck*), lipid uptake (*Cd36*), and circadian function (*Clock*) from livers of REV-ERBβ-deficient or wild-type littermate controls. (n = 7 mice per group) Data representative of 3 independent experiments demonstrating similar results. **(D)** qRT-PCR analysis of genes involved in glucose metabolism (*G6pase*, *Pepck*), lipid uptake (*Cd36*), and circadian function (*Clock*) from livers of REV-ERBα-deficient or wild-type littermate controls. For REV-ERBß KO mice: (n = 7 mice per group) Data representative of 3 independent experiments demonstrating similar results. For REV-ERBa KO mice: (n = 5 mice per group) Data representative of 2 independent experiments demonstrating similar results. Values are mean±s.e.m. Statistical significance was assessed using Student’s two-tailed *t-*tests. *p<0.05.
(TIFF)

S4 Fig REV-ERBβ-deficient mice consume more food than WT littermate controls. **(A)** Body weight and body composition of the mice in the metabolic chambers experiment. **(B)** Measurement of accumulated food intake of chow-fed, male WT and REV-ERBβ KO mice during their time in the metabolic chambers. **(C)** Analysis of food intake for 12-hour periods (day versus night) in male WT and REV-ERBβ KO mice during their time in the metabolic chambers. **(D)** Table indicating the average daily food intake, number of bouts, and % time in each bout for WT and REV-ERBβ KO mice in the BioDAQ experiments. **(E)** Body weight and body composition analysis of male mice from Fig 4 at 30 weeks of age. (n = 7 mice per group). Similar results were observed in separate cohorts of male and female mice. Values are mean±s.e.m. Statistical significance was assessed using Student’s two-tailed *t-*tests. *p<0.05.
(TIFF)
