# Supplementary figures and images for: Distinct roles for REV-ERBα and REV-ERBβ in oxidative capacity and mitochondrial biogenesis in skeletal muscle
Source: PLoS One. 2018 May 3;13(5):e0196787. doi: 10.1371/journal.pone.0196787 (PMC5933789; doi:10.1371/journal.pone.0196787)

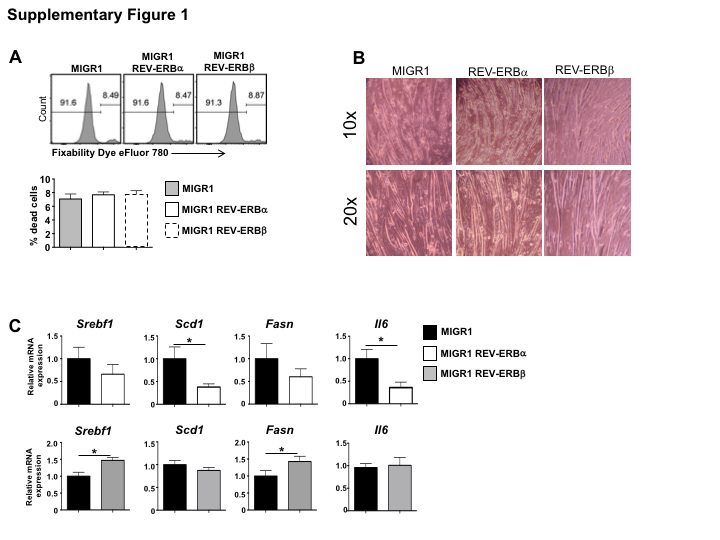

Supplement: S1 Fig — (A) Flow cytometry (FACS) after 6 days in culture using a viability dye. FACS panels (top) are representative plots of the overall health of the C2C12 cultures on Day 6. Graph (bottom) represents the average viability across all cultures per condition (n = 3). (B) Representative images of the C2C12 cultures at 10x and 20x magnification on Day 6 demonstrating the differentiation of the cells and no overt cell death. (C) qRT-PCR analysis of genes involved in lipid metabolism and inflammation, including Srebf1, Scd1, Fasn, and Il6 in C2C12 cells overexpressing REV-ERBa (top) or REV-ERBß (bottom) relative to empty vector control. Data are representative of 3 separate experiments demonstrating similar results. Statistical significance was assessed using Student’s two-tailed t-tests. *p<0.05. (TIFF) [file pone.0196787.s001.tiff]

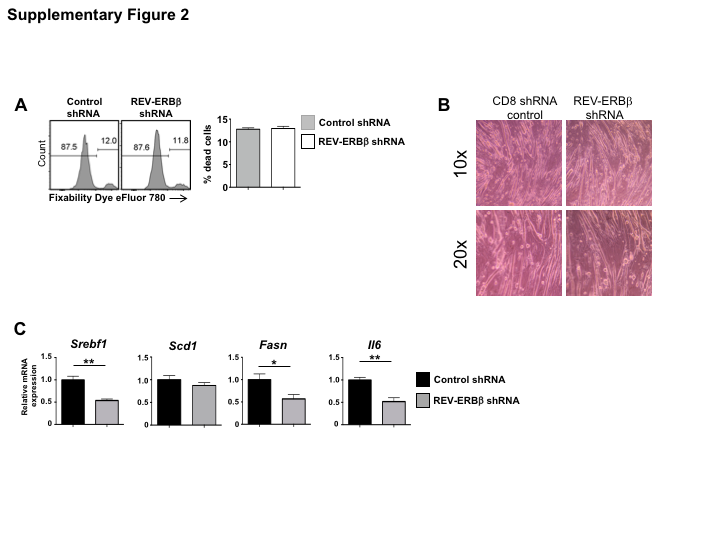

Supplement: S2 Fig — (A) Cell viability determined by flow cytometry (FACS) after 6 days in culture using a viability dye. FACS panels (top) are representative plots of the overall health of the C2C12 cultures on Day 6. Graph (bottom) represents the average viability across all cultures per condition (n = 3). (B) Representative images of the C2C12 cultures at 10x and 20x magnification on Day 6 demonstrating the differentiation of the cells and no overt cell death. (C) qRT-PCR analysis of genes involved in lipid metabolism and inflammation, including Srebf1, Scd1, Fasn, and Il6 in C2C12 cells in which REV-ERBβ was knocked down relative to CD8 control. Data are representative of 3 separate experiments demonstrating similar results. Statistical significance was assessed using Student’s two-tailed t-tests. *p<0.05. (TIFF) [file pone.0196787.s002.tiff]

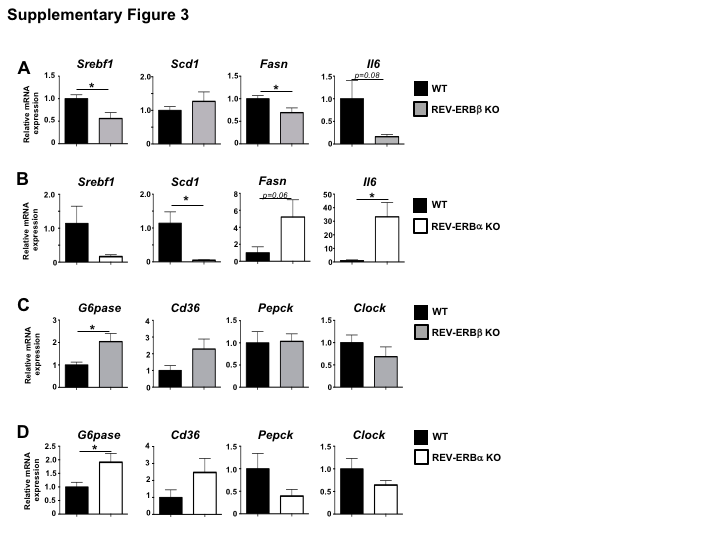

Supplement: S3 Fig — (A) qRT-PCR analysis of muscle (quadriceps) from REV-ERBβ-deficient or wild-type littermate controls. Analysis includes effects on expression of genes involved in lipid metabolism and inflammation, including Srebf1, Scd1, Fasn, and Il6. (B) qRT-PCR analysis of muscle (quadriceps) from REV-ERBα-deficient or wild-type littermate controls. Analysis includes effects on expression of genes involved in lipid metabolism, including Srebf1, Scd1, Fasn, and Il6. (C) qRT-PCR analysis of genes involved in glucose metabolism (G6pase, Pepck), lipid uptake (Cd36), and circadian function (Clock) from livers of REV-ERBβ-deficient or wild-type littermate controls. (n = 7 mice per group) Data representative of 3 independent experiments demonstrating similar results. (D) qRT-PCR analysis of genes involved in glucose metabolism (G6pase, Pepck), lipid uptake (Cd36), and circadian function (Clock) from livers of REV-ERBα-deficient or wild-type littermate controls. For REV-ERBß KO mice: (n = 7 mice per group) Data representative of 3 independent experiments demonstrating similar results. For REV-ERBa KO mice: (n = 5 mice per group) Data representative of 2 independent experiments demonstrating similar results. Values are mean±s.e.m. Statistical significance was assessed using Student’s two-tailed t-tests. *p<0.05. (TIFF) [file pone.0196787.s003.tiff]

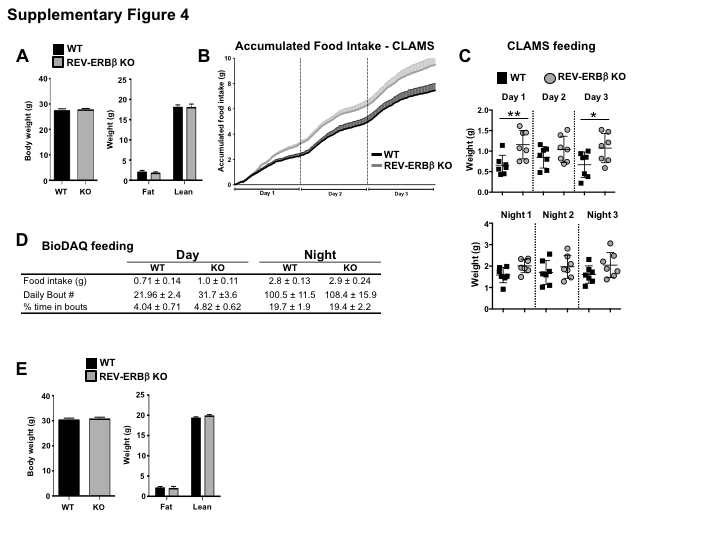

Supplement: S4 Fig — (A) Body weight and body composition of the mice in the metabolic chambers experiment. (B) Measurement of accumulated food intake of chow-fed, male WT and REV-ERBβ KO mice during their time in the metabolic chambers. (C) Analysis of food intake for 12-hour periods (day versus night) in male WT and REV-ERBβ KO mice during their time in the metabolic chambers. (D) Table indicating the average daily food intake, number of bouts, and % time in each bout for WT and REV-ERBβ KO mice in the BioDAQ experiments. (E) Body weight and body composition analysis of male mice from Fig 4 at 30 weeks of age. (n = 7 mice per group). Similar results were observed in separate cohorts of male and female mice. Values are mean±s.e.m. Statistical significance was assessed using Student’s two-tailed t-tests. *p<0.05. (TIFF) [file pone.0196787.s004.tiff]
